# Clinical and genetic features of infancy-onset congenital myopathies from a Chinese paediatric centre

**DOI:** 10.1186/s12887-021-03024-0

**Published:** 2022-01-26

**Authors:** Yu Zhang, Hui Yan, Jieyu Liu, Huifang Yan, Yinan Ma, Cuijie Wei, Zhaoxia Wang, Hui Xiong, Xingzhi Chang

**Affiliations:** 1 Department of Paediatrics, Peking University First Hospital, No.1 Xianmen Street, Xicheng District, 100034 Beijing, PR China; 2 Department of Paediatrics, Peking University International Hospital, 102206 Beijing, PR China; 3 Department of Central Laboratory, Peking University First Hospital, 100034 Beijing, PR China; 4 Department of Neurology, Peking University First Hospital, 100034 Beijing, PR China

**Keywords:** Congenital myopathy, Genetics, Infancy onset, Follow-up, Muscle biopsy

## Abstract

**Background:**

Congenital myopathies are a group of rare neuromuscular diseases characterized by specific histopathological features. The relationship between the pathologies and the genetic causes is complex, and the prevalence of myopathy-causing genes varies among patients from different ethnic groups. The aim of the present study was to characterize congenital myopathies with infancy onset among patients registered at our institution.

**Method:**

This retrospective study enrolled 56 patients based on the pathological and/or genetic diagnosis. Clinical, histopathological and genetic features of the patients were analysed with long-term follow-up.

**Results:**

Twenty-six out of 43 patients who received next-generation sequencing had genetic confirmation, and *RYR1* variations (12/26) were the most prevalent. Eighteen novel variations were identified in 6 disease-causing genes, including *RYR1*, *NEB, TTN, TNNT1, DNM2* and *ACTA1.* Nemaline myopathy (17/55) was the most common histopathology. The onset ages ranged from birth to 1 year. Thirty-one patients were followed for 3.83 ± 3.05 years (ranging from 3 months to 11 years). No patient died before 1 year. Two patients died at 5 years and 8 years respectively. The motor abilities were stable or improved in 23 patients and deteriorated in 6 patients. Ten (10/31) patients developed respiratory involvement, and 9 patients (9/31) had mildly abnormal electrocardiograms and/or echocardiograms.

**Conclusion:**

The severity of congenital myopathies in the neonatal/infantile period may vary in patients from different ethnic groups. More concern should be given to cardiac monitoring in patients with congenital myopathies even in those with static courses.

**Supplementary Information:**

The online version contains supplementary material available at 10.1186/s12887-021-03024-0.

## Introduction

Congenital myopathies are a group of rare genetic muscle disorders characterized clinically by generalized hypotonia and weakness, which generally occur from birth, and a static or slowly progressive course [1–3]. The classification of congenital myopathies is based mainly on the features observed in muscle biopsies. Accordingly, congenital myopathies are divided into the following forms: nemaline myopathy (NM), core myopathy, centronuclear myopathy (CNM), congenital fibre type disproportion (CFTD), and myosin storage myopathy [2]. To date, variations in at least 27 genes have been reported to cause congenital myopathy. Due to the use of next-generation sequencing, the number of variations related to congenital myopathy is expected to increase [4]. However, the relationship between the pathologies and the genetics of congenital myopathies is complex. Multiple genes can cause the same pathology, and variations in the same gene may cause different histopathologies. For example, CNM has been associated with at least seven genes, including *MTM1*, *DNM2*, *RYR1*, *TTN, BIN1*, *CCDC78* and SPEG [4], whereas *RYR1* variations can cause central core disease (CCD) [5, 6], multiminicore disease (MmD) [7], core-rod myopathy [8], CNM [9] and CFTD [7]. In addition, patients with different pathological changes may present with similar clinical manifestations.

A static or slowly progressive course is recognized as a key clinical feature of congenital myopathies. Most patients with congenital myopathies will survive into adulthood, except when serious respiratory insufficiency and severe weakness are present in childhood [2, 3]. Mortalities from congenital myopathies vary in different cohorts of patients [10–12]. For many neuromuscular diseases, cardiac involvement represents a major cause of morbidity and mortality [13]. However, the major cause of death in patients with congenital myopathies is respiratory failure. Except for a few cases related to *TTN* [14], *MHY7* [15, 16] and *FLNC* [17] variations, patients with congenital myopathies usually have no or only mild cardiac affliction [18].

Using next-generation sequencing (NGS) techniques, many new genes are being identified as the causal genes of congenital myopathies as well as new variations identified in known genes, broadening the genotype spectrum of congenital myopathies [3]. The prevalence of myopathy-causing genes varies among patients from different ethnic groups. For example, variations in *KLHL*40 are a frequent cause of severe NM in Japanese patients [19], while *TNNT1* variations are common among Amish people with severe NM [20]. Based on current knowledge of congenital myopathies, we retrospectively investigated 56 cases of infancy-onset congenital myopathies, including the clinical, pathological and genetic characteristics as well as long-term follow-up data. The present study emphasized infantile mortality and functional outcomes, combined with gene diversity.

## Patients and methods

### Patients

Fifty-six patients with congenital myopathy (CM) who were registered from January 2007 to December 2019 (inclusive) in the Department of Paediatrics, Peking University First Hospital were included in this study. Patients with an infancy onset were defined as those exhibiting motor developmental delays in no more than 1 year of age. The clinical manifestation of the enrolled patients was consistent with the general presentation of congenital myopathies as follows: generalized muscle weakness and hypotonia; normal or mildly elevated serum creatine kinase (CK); and normal or mild myopathic changes on electromyogram examination. In addition, the patients had either characteristic histopathological features of congenital myopathies, definitive genetic diagnosis or both. One case (Pt 3) was previously reported in our case report in 2013, and clinical manifestation and family segregation were presented without follow-up [21]. NM patients (Pt 10–11 and Pt 35–38) were included in one of our previous studies focused on the genetic features of NM patients with onset ages ranging from infancy to adulthood [22]. Open muscle biopsies were performed in 55 patients (98.2%, one patient refused), and pathological diagnosis was made according to the criteria suggested by Dubowitz et al. [23]. Genetic analysis was performed in 43 patients (some patients diagnosed at earlier times when commercial NGS was not available were lost to follow-up), and 31 patients were followed up. Written informed consent was obtained from all patients and/or their parents prior to inclusion in the study. The present study was approved by the Ethics Committee of Peking University First Hospital (Beijing, China, Approved number: 2018–265).

### Muscle biopsy

Open muscle biopsies were taken from the biceps or quadriceps femoris. Fresh frozen muscle specimens were fixed in liquid nitrogen, and frozen sections (8–10 μm) were processed. A series of histochemical methods was used in all muscle specimens, including haematoxylin and eosin (H&E), modified Gomori trichrome (MGT), oil Red O, periodic acid-Shiff (PAS), nicotinamide adenine dinucleotide dehydrogenase-tetrazolium reductase (NADH-TR), succinate dehydrogenase (SDH), cytochrome c oxidase (COX), nonspecific esterase and adenosine triphosphatase (ATPase) staining. The ultrastructure was observed under transmission electron microscopy (JEM1230, Japan). The pathological categories were classified according to the criteria suggested by Dubowitz et al. [23]: (1) core myopathies including CCD and MmD; (2) NM; (3) CNM; (4) CFTD; and (5) nonspecific myopathic changes (NSMC). We included patients with mixed structural pathological features, for example, a combination of cores and rods. However, we did not consider type 1 fibre dominance/uniformity as an isolated diagnosis due to its low specificity and potential coexistence with other more specific features.

### Gene variation analyses

Blood samples were collected, and genomic DNA was extracted from leukocytes of patients. The coding exons of the 169 myopathy-causing genes (Supplement 1) were selected by a gene capture strategy (Kangso Medical Inspection, China; MyGenostics, Beijing). The gene panel was used to screen for variations causing hereditary myopathies, and high coverage (≥100 ×) sequencing was used to detect single nucleotide polymorphisms (SNPs) and indel variations. The variations taken into further consideration were as follows: (1) marked as a pathogenic variation in the Human Genome Mutation Database (HGMD); (2) nonsense variations; (3) frameshift variations; and (4) located in an essential splice site. The pathogenicity of nonsynonymous variation was evaluated by using multiple algorithms, including SIFT, Ployphen-2, Mutation-Taster, PROVEAN and Splice-Site Prediction by Neural Network. When pathogenic variations were not found in some patients through panel screening, whole exome sequencing was used for further study. The exomes were captured using the xGen Exome Research Panel v1.0 (Integrated DNA Technologies) and sequenced on an Illumina HiSeq2000 (Illumina, San Diego, CA, USA) with 100-bp paired-end reads (Kangso Medical Inspection, China; MyGenostics, Beijing). After the sequencing, the raw data were saved as a FASTQ format, and then we did the bioinformatics analysis. First, Illumina sequencing adapters and low-quality reads (< 80 bp) were filtered by cut adapt. After quality control, the clean reads were mapped to the UCSC hg19 human reference genome using BWA. Duplicated reads were removed using picard tools, and mapping reads were used for variation detection. Second, the variations of SNP and InDel were detected by GATK Haplotype Caller, then we used GATK Variant Filtration to filter variations. The filtered standards were as follows: a) variations with mapping qualities < 30; b) the Total Mapping Quality Zero Reads < 4; c) approximate read depth < 5; d) QUAL< 50.0; e) Phred-scaled *p*-value using Fisher’s exact test to detect strand bias > 10.0. After above two steps, the data would be transformed to VCF format. Variations were further annotated by ANNOVAR and associated with multiple databases, such as 1000 genome, ESP6500, dbSNP, EXAC, Inhouse (MyGenostics), HGMD, as well as predicted by SIFT, PolyPhen-2, MutationTaster, GERP++.Then four steps were used to select the potential pathogenic variations in downstream analysis: (i) variation reads should be more than 5, variation ration should be no less than 30%; (ii) removing the variations with frequency of more than 5% in 1000 genome, ESP6500 and Inhouse database; (iii) If the variations existed in InNormal database (MyGenostics), then dropped; (iv) the synonymous variations reported in HGMD were left, and the remaining of the synonymous variations were excluded. All candidate pathogenic variations were confirmed by Sanger sequencing. The interpretation of the variations was made according to the guidelines provided by the American College of Medical Genetics (ACMG) [24].

### Patient follow-up

Patients were followed up at our outpatient department. Mobility and musculoskeletal complications were evaluated with physical examinations. The manual muscle test (MMT) was used to assess the degree of muscle weakness. Posteroanterior radiographs were performed in selected patients with suspected scoliosis, and a Cobb angle of more than 10° was considered significant. Mild scoliosis was defined as a Cobb angle ranging from 10° to 40°, and severe scoliosis was defined as a Cobb angle over 40°. Forced vital capacity (FVC) was measured and expressed as a percentage of the predicted value for height in patients over 6 years old. Cardiac function was monitored with electrocardiograms (ECGs) and echocardiograms.

## Results

### Gene variations

Pathogenic variations were identified in 26 out of the 43 patients (26/43,60.5%). In total, 40 pathogenic and likely pathogenic variants were confirmed in 6 myopathy-related genes (Table 1 and Supplement 2). Of them, 18 variants were novel, including eight *RYR1* variations (c.3880G > T, c.658C > T, c.4715 T > C, c.4454G > A, c.2044C > G, c.6823G > A, c.1675dup and c.7330C > T), two *NEB* variations *(*c.3567 + 1G > A and c.6734dup), one *ACTA1* variation *(*c.402G > T), one *DNM2* variation (c.1893 + 1G > A), four *TTN* variations (c.32312-1G > A, c.2099_2106dup, c.85818 T > A and c.102798-102800del), and two *TNNT1* variations (c.1A > G and c.353delC). Variations in *RYR1* were identified in 12 patients (12/43, 27.8%), being the most frequent gene variations in both dominant and recessive inheritance. All dominant *RYR1* variations were pathologically associated with CCD, and recessive variations were associated with different pathological changes (MmD, 3 cases; CFTD, 1 case; and CNM 1 case). Variations associated with NM were identified in three genes, including compound heterozygous *NEB* variations in 4 patients (4/43, 9.3%), heterozygous *ACTA1* variations in 4 patients (4/43, 9.3%) and compound heterozygous *TNNT1* variations in one patient (1/43, 2.3%). Compound heterozygous *TTN* variations were found in three patients (3/43, 7%) with different pathological diagnoses (MmD, 2 cases; and CNM, 1 case). Heterozygous *DNM2* variations were found in 2 patients (2/43, 4.7%) with CNM.

**Table 1:** Variations identified in the patients with congenital myopathies

Case	Pathology	Gene	Inheritance	Variations	Protein	Origin	Pathogenicity	ACMG criterion	PMID
1	CCD	*RYR1*	AD	c.14596A > G^‡^	p.(Lys4866Gln)	De novo	Pathogenic	PS2 + PM1 + PM2 + PP3 + PP4	25,331,388
2	CCD	*RYR1*	AD	c.7111G > A^‡^	p.(Glu2371Lys)	De novo	Pathogenic	PS1 + PS2 + PM1 + PM2 + PP3 + PP4 + PP5	29,293,505
3^#^	CCD	*RYR1*	AD	c.14678 G > A^‡^	p.(Arg4893Gln)	Paternal	LP	PM1 + PM2 + PP3 + PP4 + PP5	12,565,913
4^#^	CCD	*RYR1*	AD	c.14741G > C^‡^	p.(Arg4914Thr)	Paternal	Pathogenic	PS1 + PM1 + PM2 + PP3 + PP4 + PP5	12,565,931
5	MmD	*TTN*	AR	c.85818 T > A	p.(Tyr28606Ter)	Paternal	Pathogenic	PVS1 + PS1 + PM2 + PP3 + PP4 + PP5	
				c.102798_102800del	p.(Asn34266del)	Maternal	LP	PM2 + PM3 + PM4 + PP4	
6	MmD	*RYR1*	AR	c.3880G > T	p.(Val1294Phe)	Maternal	LP	PM1 + PM2 + PP3 + PP4 + PP5	
				c.14473C > T^‡^	p.(Arg4825Cys)	Paternal	Pathogenic	PS1 + PM1 + PM2 + PP3 + PP4 + PP5	20,301,565
7^#^	MmD	*RYR1*	AR	c.658C > T	p.(Arg220Cys)	Paternal	LP	PM1 + PM2 + PP1 + PP3 + PP4 + PP5	
				c.4715 T > C	p.(Met1572Thr)	Maternal	LP	PM1 + PM2 + PP1 + PP3 + PP4 + PP5	
8	MmD	*RYR1*	AR	c.4454G > A	p.(Ser1485Asn)	Paternal	LP	PM1 + PM2 + PP3 + PP4 + PP5	
				c.3494G > A^‡^	p.(Gly1165Asp)	Maternal	Pathogenic	PS1 + PM1 + PM2 + PP3 + PP4	21,911,697
9	NM	*TNNT1*	AR	c.1A > G	p.?	Maternal	Pathogenic	PVS1 + PM2 + PP4 + PP5	
				c.353delC	p. (Thr118Metfs Ter16)	Paternal	Pathogenic	PVS1 + PM2 + PP4	
10	NM	*NEB*	AR	c.18808C > T^‡^	p.(Arg6270Ter)	Maternal	Pathogenic	PVS1 + PS1 + PM2 + PP3 + PP4 + PP5	25,205,138
				c.2311-2A > C^‡^	p.?	Paternal	Pathogenic	PVS1 + PM2 + PP4	32,222,963
12	CNM	*TTN*	AR	c.2099_2106dup	p.(Ala703LysfsTer3)	Maternal	Pathogenic	PVS1 + PS1 + PM2 + PM3 + PP4	
				c.107377 + 1G > A^‡^	p.?	Paternal	Pathogenic	PVS1 + PS1 + PM2 + PP4	25,589,632
13	CNM	RYR1	AR	c.6823G > A	p. (Val2275Met)	Maternal	LP	PM1 + PM2 + PP3 + PP4 + PP5	
				c.2044C > G	p.(Arg682Gly)	Paternal	LP	PM1 + PM2 + PM5 + PP3 + PP4 + PP5	
14	CNM	*TTN*	AR	c.95341C > T^‡^	p.(Arg31781Ter)	De novo	Pathogenic	PVS1 + PS2 + PM2 + PP3 + PP4	25,163,546
				c.32312-1G > A	p.?	Maternal	Pathogenic	PVS1 + PS1 + PM2 + PP4	
15	CNM	*DNM2*	AD	c.1893 + 1G > A	p.?	De novo	Pathogenic	PVS1 + PS1 + PS2 + PM2 + PP4	
16	CNM	*DNM2*	AD	c.1856C > T^‡^	p.(Ser619Leu)	De novo	Pathogenic	PS1 + PS2 + PM1 + PM2 + PP2 + PP3 + PP4 + PP5	32,860,008
17	CFTD	*RYR1*	AR	c.12536G > A^‡^	p.(Arg4179His)	Maternal	Pathogenic	PS1 + PM1 + PM2 + PP3 + PP4	21,062,345
				c.1675dup	p.(Ile559AsnfsTer11)	Paternal	Pathogenic	PVS1 + PM2 + PS1 + PP4	
18	/	*RYR1*	AR	c.3523G > A^‡^	p.(Glu1175Lys)	Maternal	LP	PM1 + PM2 + PP3 + PP4	25,635,128
				c.7330C > T	p.(Gln2444Ter)	Paternal	Pathogenic	PVS1 + PS1 + PM2 + PP4 + PP5	
34	CCD	*RYR1*	AD	c.14447A > G^‡^	p.(Asp4816Gly)	De novo	Pathogenic	PS1 + PS2 + PM1 + PM2 + PM5 +PP3 + PP4 + PP5	23,553,484
35	CCD	*RYR1*	AD	c.14582G > A^‡^	p.(Arg4861His)	De novo	Pathogenic	PS1 + PS2 + PM1 + PM2 + PP3 + PP4	25,521,991
36^#^	NM	*NEB*	AR	c.3567 + 1G > A	p.?	Paternal	Pathogenic	PVS1 + PS1 + PM2 + PP3 + PP4	
				c.6734dupA	p. (Thr2246Aspfs Ter8)	Maternal	Pathogenic	PVS1 + PM2 + PP3 + PP4	
37	NM	*NEB*	AR	c.19944G > A^‡^	p.?	Maternal	LP	PS1 + PM2 + PP3 + PP4	25,205,138
				c.6029del^‡^	p. (Ile2010Thrfs Ter14)	Paternal	Pathogenic	PVS1+ PM2 + PP4	32,222,963
38	NM	*NEB*	AR	c.7818delG^‡^	p. (Met2606Ilefs Ter13)	Paternal	Pathogenic	PVS1+ PM2 + PP4	32,222,963
				c.24579G > A ^‡^	p.?	Maternal	LP	PS1 + PM2 + PP3 + PP4 + PP5	24,725,366
39	NM	*ACTA1*	AD	c.400A > G^‡^	p.(Met134Val)	De novo	Pathogenic	PS1 + PS2 + PM1 + PM2 + PP2 + PP3 + PP4 + PP5	10,508,519
40	NM	*ACTA1*	AD	c.515C > G^‡^	p.(Ala172Gly)	De novo	Pathogenic	PS2 + PM2 + PM5 + PP2 + PP3 + PP4	12,921,789
41	NM	*ACTA1*	AD	c.402G > T	p.(Met134Ile)	De novo	Pathogenic	PS1 + PS2 + PM1 + PM2 + PP2 + PP3 + PP4 + PP5	
42	NM	*ACTA1*	AD	c.109G > T^‡^	p.(Val37Leu)	De novo	Pathogenic	PS1 + PS2 + PM1 + PM2 + PP2 + PP3 + PP4 + PP5	25,326,635

*Note*: Reference transcript of different genes: *RYR1*, NM_000540.2; *NEB*, NM_001164507.1; *DNM2*, NM_001005360.2; *ACTA1*, NM_001100.3; *TNNT1*, NM_001126132.1; *TTN*, NM_001267550.1. PMID: PubMed Unique Identifier

*Abbreviations*: *#* Positive family history, *‡* Previously reported, / data unavailable, *CCD* Central core disease, *MmD* Multiminicore disease, *NM* Nemaline myopathy, *CNM* Centronuclear myopathy, *CFTD* Congenital muscle fiber disproportion, *AD* Autosomal dominant, *AR* Autosomal recessive, *ACMG* American College of Medical Genetics, *LP* Likely pathogenic

### Pathologic characteristics

**Fig. 1:**
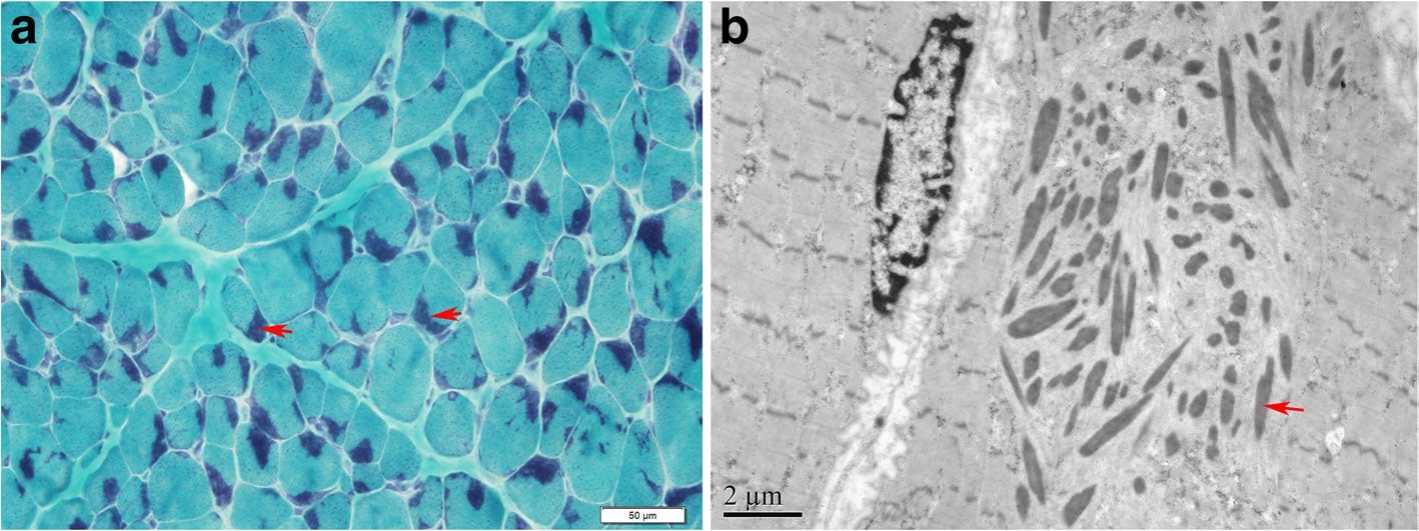
Nemaline rods in NM patients (**A**, patient 46, right quadriceps femoris). Transverse sections of muscle specimens stained with modified Gomori trichrome show numerous nemaline rods (arrows) clustered under the sarcolemmal membrane in muscle fibres. (**B**, patient 46, right quadriceps femoris) Electron microscopy shows many typical rods with disorganized myofibrils, and the rods have the same electron density as the Z-disk (arrow)

**Fig. 2:**
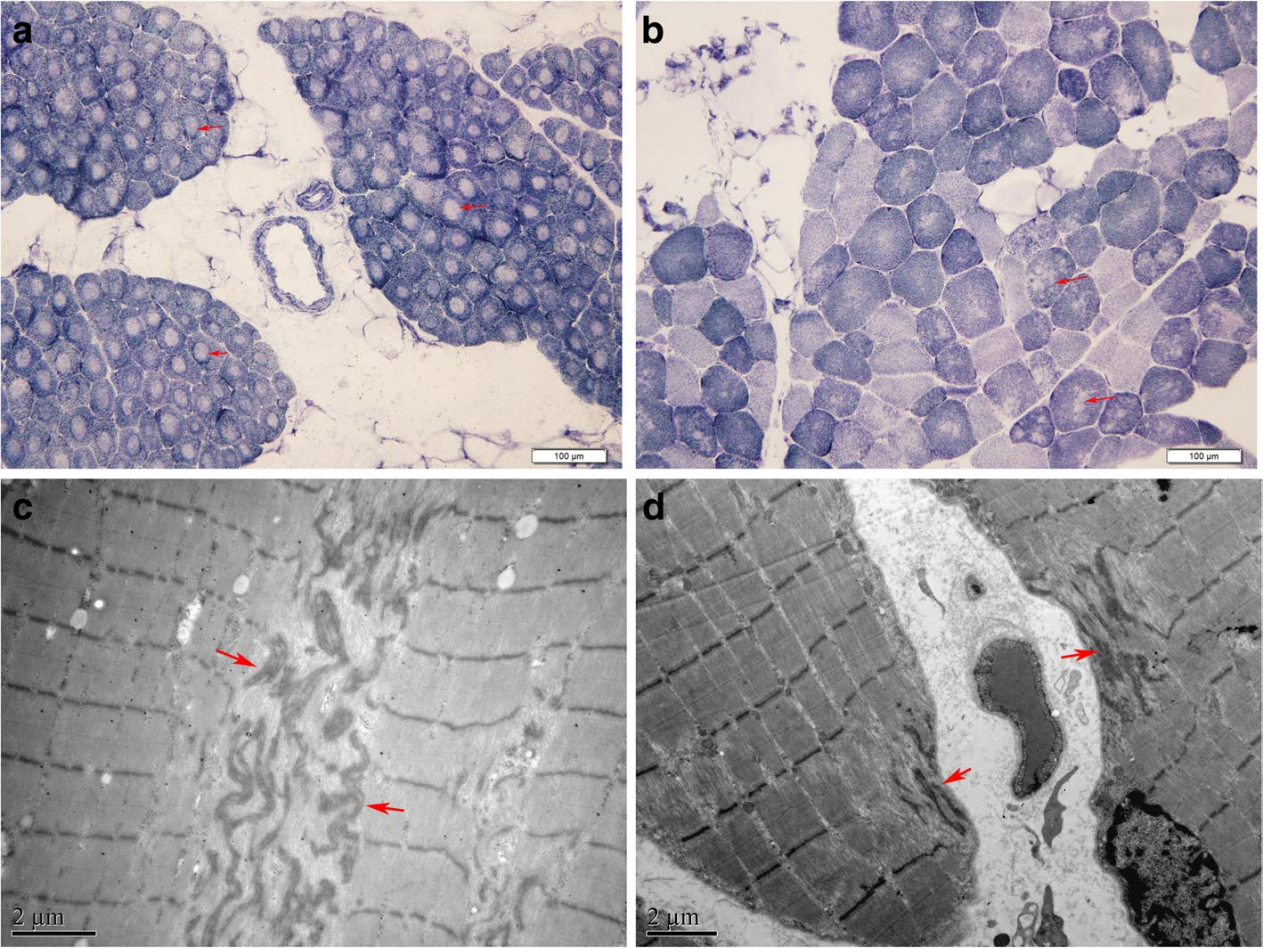
Central cores and minicores in patients with core myopathies. (**A**, patient 1, left biceps) Reduced nicotinamide adenine dinucleotide–tetrazolium reductase (NADH-TR) staining of transverse sections of muscle specimens shows the typical central cores. The cores are single, central placed, well-circumscribed, circular regions (arrows) that appeared in almost all fibres. (**B**, patient 6, right quadriceps femoris) Minicores are demonstrated as multiple foci of ill-defined small areas with deficiency of oxidative activity on NADH-TR staining (arrows). (**C**, patient 1, left biceps) Electron microscopy shows that the central cores (arrows) contain disorganized myofibrils with Z-line streaming. (**D**, patient 6, right quadriceps femoris) Electron microscopy shows two minicores (arrows) at the periphery of muscle fibres

**Fig. 3:**
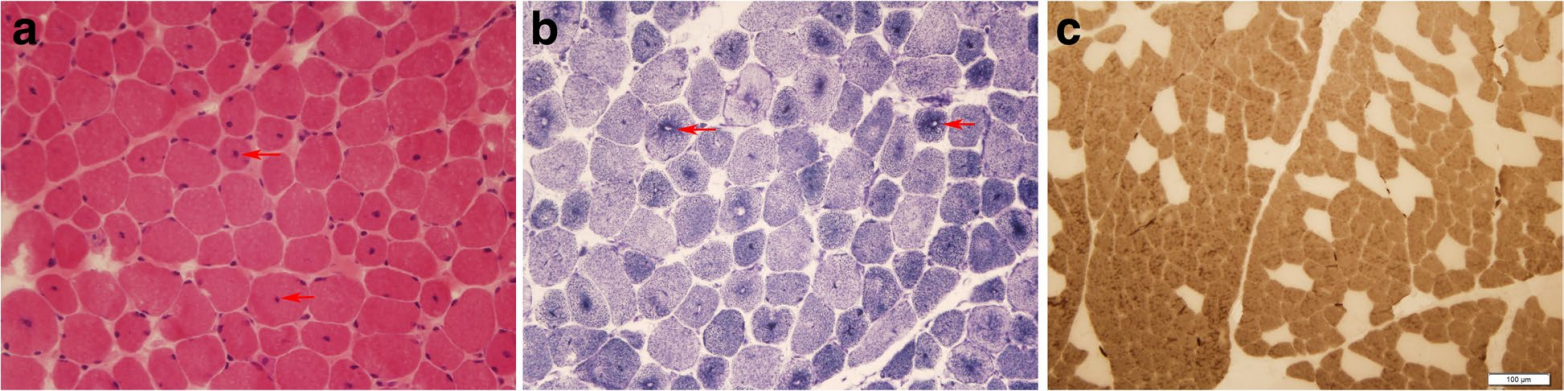
Transverse sections of muscle specimens from patients with CNM and CFTD. (**A**, patient 15, left biceps) Central nuclei (arrows) appeared in more than 30% of fibres (haematoxylin and eosin staining). (**B** patient 15, left biceps) The intermyofibrillar network revealed by reduced nicotinamide adenine dinucleotide–tetrazolium reductase (NADH-TR) staining, showing radiates like spokes of a wheel from the centre to the periphery of the fibres (arrows). (**C**, patient 17, left biceps) Adenosine triphosphatase (ATPase) staining with preincubation at pH 4.6. Type 1 fibers (dark type) are at least 12% smaller than type 2 fibres (pale type) accompanied by type 1 fibre predominance

A total of 55 muscle specimens were collected. NM (30.9.8%, 17/55) was the most frequent histopathological diagnosis in this cohort followed by core myopathy (29.1%, 16/55). Typical nemaline rods (Fig. 1) were the main pathological findings in 17 patients. Central cores (Fig. 2A, C) were observed in 10 patients, and multiple minicores (Fig. 2B, D) were noted in 6 patients. The coexistence of cores and rods was observed in only one patient (Pt 19). Characteristic changes of CNM were found in 10 patients (18.2%, 10/55), including central nuclei in a large number of muscle fibres (> 30%), associated with radial arrangements of sarcoplasmic strands in patients with *DNM2* mutations (Fig. 3A, B). CFTD was diagnosed in 11 patients (20%, 11/55). The key pathological change was the presence of type 1 fibres that were at least 12% smaller than type 2 fibres without other significant histological abnormalities (Fig. 3C).

### Clinical features

The enrolled 56 patients with congenital myopathies included 32 boys and 24 girls (Table 2), and they were diagnosed as follows: 25 patients were diagnosed by characteristic pathological features and pathogenic gene variations; 30 patients were diagnosed by typical histopathologic features; and one patient was genetically diagnosed without muscle pathology. We followed up with 31 (31/56, 55.4%) patients for 3.83 ± 3.05 years (ranging from 3 months to 11 years) (Table 2). Seven patients had a significant family history, which suggested that the inheritance pattern was autosomal dominant in 3 patients and autosomal recessive in 4 patients. Premature death in family members was noted in 4 families. There was no history of parental consanguinity and no history of miscarriage or abortion in the family.

**Table 2:** Clinical, pathological and genetic data of 58 patients

Case/FH	Sex	Age at onset (m)/diagnosis(y)	Weakness pattern	maximum motor/(m)	Gene	Pathology	Follow-up age (y)	Motor function	Pulmonary function	Cardiac involvement	Contractures/ Tightness of Archilles
1/−	F	6/6	Generalized	Walk/24	*RYR1*	CCD	17	Wheelchair	N	N	−/+
2/−	F	Birth/1.5	Generalized	Sit/18	*RYR1*	CCD	3	Sit	N	N	+/+
3/AD	F	12/16	Generalized-	Walk/18	*RYR1*	CCD	18	Ambulation	N	N	−/−
4/AD	M	Birth/3	Generalized	Walk/36	*RYR1*	CCD	12	Wheelchair	N	N	−/+
5/−	M	12/6	Generalized	Walk/24	*TTN*	MmD	11	Ambulation	N	A	−/+
6/−	M	12/9	Generalized	Walk/14	*RYR1*	MmD	13	Ambulation	N	N	−/−
7/AR	M	Birth/0.4	Generalized	Head control/5	*RYR1*	MmD	2.5	Ambulation	N	A	−/−
8/−	M	Birth/12	Generalized	Walk/36	*RYR1*	MmD	14.3	Ambulation	N	A	−/+
9/−	F	6/2.6	Axial/ Generalized	Walk/24	*TNNT1*	NM	11	Cane	A (FEV35.6%)	A	+/+
10/−	M	2/4.1	Generalized	Walk/24	*NEB*	NM	9	Ambulation	A (FEV32.1%)	N	−/−
11/−	M	4/3	Generalized	Walk/18	unknown	NM	7	Ambulation	N	N	−/+
12/−	F	12/5	Generalized	Walk/19	*TTN*	CNM	13	Ambulation	N	N	−/+
13/−	M	12/12	Generalized	Walk/16	*RYR1*	CNM	18	Ambulation	N	N	−/+
14/−	F	4/2	Generalized	Aided standing /24	*TTN*	CNM	4	Ambulation	N	A	−/−
15/−	M	12/6	Distal	Walk/17	*DNM2*	CNM	6.8	Ambulation	N	N	−/+
16/−	M	Birth/7	Generalized	Walk/13	*DNM2*	CNM	9.6	Ambulation	N	N	−/−
17/−	F	12/13	Generalized	Walk/24	*RYR1*	CFTD	17.2	Ambulation	N	N	−/−
18/−	M	Birth/12	Generalized	Walk/13	*RYR1*	/	13	Ambulation	N	N	−/−
19/−	F	8/9	Axial/Generalized	Walk/14	Unknown	MmD/NM	10.1	Cane	Intermittent ventilation	N	+/+
20/AR	F	Birth/8	Generalized	Walk/16	Unknown	MmD	9.1	Ambulation	Intermittent ventilation	A	−/+
21/−	M	Birth/10.8	Generalized	Walk/24	Unknown	NM	11.3	Ambulation	N	N	−/−
22/−	F	12/13	Generalized	Walk/18	Unknown	NM	16	Ambulation	A (FEV30.7%)	A	−/+
23/−	F	Birth/8	Generalized	Walk/18	Unknown	NM	9.1	Ambulation	A (FEV46%)	N	−/−
24/AR	F	12/2.3	Generalized	Sit/27	Unknown	NM	3.3	Sit	N	N	−/−
25/−	M	12/4	Generalized	Walk/24	Unknown	CNM	10	Ambulation	A (FEV72.5%)	N	+/+
26/−	M	3/5	Generalized	Walk/48	Unknown	CNM	8	Cane/died	Continuous ventilation	A	+/+
27/−	F	Birth/1.9	Axial / Generalized	Aided walking/22	Unknown	CFTD	8	Wheelchair	A	N	−/+
28/−	M	4/4	Generalized	Walk/24	/	CFTD	5	Ambulation/died	Continuous ventilation	N	−/−
29/−	M	Birth/8	Generalized	Walk/20	Unknown	CFTD	14	Ambulation	N	N	+/+
30/−	M	12/2.7	Generalized	Walk/16	/	CFTD	12.7	Ambulation	N	N	−/−
31/−	M	12/6.8	Generalized	Walk/13	Unknown	CFTD	7	Ambulation	N	A	−/−
32/−	M	Birth/3.8	Generalized	Walk/19	*RYR1*	CCD	/	/	/	/	/
33/−	F	Birth/2	Generalized	Walk/13	*RYR1*	CCD	/	/	/	/	/
34/AD	F	4/3	Generalized	Walk/30	*NEB*	NM	/	/	/	/	/
35/−	F	Birth/3	Generalized	Walk/18	*NEB*	NM	/	/	/	/	/
36/−	M	Birth/3.8	Generalized	Walk/18	*NEB*	NM	/	/	/	/	/
37/−	M	12/5	Generalized	Walk/14	*ACTA1*	NM	/	/	/	/	/
38/−	F	Birth/6	Generalized	Walk/24	*ACTA1*	NM	/	/	/	/	/
39/−	M	4/2.1	Generalized	Walk/22	*ACTA1*	NM	/	/	/	/	/
40/−	F	12/5	Generalized	Walk/15	*ACTA1*	NM	/	/	/	/	/
41/−	M	12/8	Generalized	Walk/16	Unknown	CCD	/	/	/	/	/
42/−	M	7/3.3	Generalized	Sit/18	/	CCD	/	/	/	/	/
43/AR	F	Birth/4	Generalized	Walk/48	/	CCD	/	/	/	/	/
44/−	F	8/7	Generalized	Walk/60	/	CCD	/	/	/	/	/
45/−	M	12/9	Generalized/	walk/13	/	MmD	/	/	/	/	/
46/−	M	8/2	Generalized	Walk/18	Unknown	NM	/	/	/	/	/
47/−	M	Birth/6	Generalized	Walk/14	Unknown	NM	/	/	/	/	/
48/−	F	12/5	Generalized	Walk/18	/	NM	/	/	/	/	/
49/−	M	Birth/13	Generalized	Walk/13	/	CNM	/	/	/	/	/
50/−	F	12/4	Generalized	Walk/18	/	CNM	/	/	/	/	/
51/−	F	Birth/2.6	Generalized	Walk/18	Unknown	CNM	/	/	/	/	/
52/−	M	12/2	Generalized	Walk/16	unknown	CFTD	/	/	/	/	/
53/−	M	Birth/1.1	Generalized	Roll/13	/	CFTD	/	/	/	/	/
54/−	M	Birth/0.8	Generalized	Head up/10	/	CFTD	/	/	/	/	/
55/−	M	Birth/1.1	Generalized	Sit/13	/	CFTD	/	/	/	/	/
56-	F	Birth/6	Generalized	Walk/14	/	CFTD	/	/	/	/	/

*Note*: /, no data, +, present; −, absent; *FH* Family history; *AD* Autosomal dominant; *AR* Autosomal recessive; *M* Male; *F* Female; N normal; A abnormal; *CCD* Central core disease; *MmD* Multiminicore disease; *NM* Nemaline myopathy; *CNM* Centronuclear myopathy; *CFTD* Congenital fiber type disproportion

Thirty-four patients (34/56, 60.7%) who had an earlier onset age of less than 6 months presented with pronounced generalized hypotonia, weakness and motor milestone delay. Eight patients (14.3%, 8/56) had bulbar involvement manifested with dysphagia and congenital laryngeal stridor, and of these patients, four had sucking failure requiring nasogastric tube feeding for up to 8 months. Two patients had respiratory insufficiency requiring short-term ventilator support (Pts 53 and 54). Twenty-two (39.3%, 22/56) patients who had an onset age over 6 months presented with climbing difficulties, abnormal gait or a tendency to fall frequently compared to their peers. In total, three patients presented with congenital hip dysplasia, and one patient had lordosis and knee contracture at the first evaluation. Reduced foetal movements were noted in three patients, and premature birth was noted in the other three patients.

The diagnostic ages ranged from 5 months to 16 years (5.53 ± 3.66 y). Although 34 patients had pronounced weakness before the age of 6 months, only 4 patients (Pts 7, 53, 54 and 55) were diagnosed before the age of 13 months. All 4 patients had severe weakness with bulbar and respiratory involvement at birth. One patient (Pt 7) had a positive family history as the elder sister of Pt 7 died shortly after birth due to pulmonary failure and severe muscle weakness.

Generalized muscle weakness, as an initial symptom, was noted in the majority of patients (55/56, 98.2%), and it was more severe in the lower limbs and more prominent in the proximal muscles. One patient (Pt 15) with centronuclear myopathy due to *DNM2* heterozygous variation presented with predominant weakness of the distal lower limbs. Three patients showed prominent axial muscle weakness from the early onset of the disease. Twenty-seven (27/56, 48.2%) patients showed facial muscle weakness, manifesting as an elongated face, high-arched palate, tented upper lip, micrognathia, nasal tone vocalization and slurred speech. Disorders of ocular motility with eyelid ptosis were found in 2 patients (Pts 2 and 54). With the development of the disease, additional involvement of axial and distal muscles was common in patients with follow-ups. Seventeen patients (17/31, 54.4%) developed variable scoliosis, and one patient (Pt 14) showed spinal rigidity. Six patients (6/31, 19.4%) demonstrated multiple joint contractures, and 17 patients (17/31, 54.8%) showed tightness of the Archilles tendon. Three patients had hypertrophy of the gastrocnemius.

Muscle weakness improved gradually in all patients during the neonatal and infancy periods. Eight patients (8/56) had bulbar involvement at onset, and four of them required nasogastric tube feeding. Bulbar impairment improved gradually, and no patient required tube feeding after 8 months of age. No gastrostomy was performed. Three patients were lost to follow-up before they gained walking ability (at 10–13 months). Forty-three (43/56, 76.8%) patients achieved independent ambulation before 2 years of age, and 7 patients achieved independent ambulation at 2.5–5 years. Moreover, 3 patients (Pts 2, 24 and 42) never achieved independent ambulation by their last visit at 3–3.3 years. Among the patients with follow-ups, 23 patients (74.2%, 23/31) remained ambulatory, but some of them had an unstable gait and experienced frequent falls. Six (6/31, 19.4%) patients gradually lost ambulation at an average age of 11.02 ± 3.05 years (ranging from 8 to 17 years), who initially acquired independent ambulation. Severity variance was noted between *RYR1* patients with dominant and recessive inheritance. Of the four patients with dominant *RYR1* variations, one (Pt 2) could only sit with support at her last visit (3 years old), one lost ambulation at 12 years, one lost ambulation at 17 years, and one could walk independently at 18 years. All six patients with recessive variations walked independently, and 5 of them were more than 13 years of age (ranging 13 to 18 years) (Table 2).

Two patients (Pts 53 and 54) with CFTD developed respiratory insufficiency, requiring continuous positive airway pressure therapy immediately after birth, but they gradually improved to discontinue the need for ventilators. No patients died within their first year of life. During follow-ups, respiratory problems were found in ten (32.3%, 10/31) patients after infancy. Two out of the patients (Pts 19 and 20) required night intermittent ventilator support (one at 8 years old and one at 9 years old). Two patients (Pts 26 and 28) died of respiratory failure at 8 and 5 years old, respectively. Five patients had decreased forced vital capacity (FVC) to 30–72.5% of expected values. One patient (Pt 27) complained of mild breathing difficulty without an FVC measurement. NM (4/10) was more frequently related to respiratory insufficiency later, and CFTD was associated with early-onset respiratory failure as the two patients with respiratory insufficiency at birth had the same CFTD.

No child complained of cardiac symptoms. Fifty-five (55/56, 98.3%) patients had normal echocardiograms with or without electrocardiogram at initial evaluation, except for one patient (Pt 45) who visited our hospital for the first time at 9 years old with respiratory insufficiency and mild pulmonary hypertension. Thirty-one patients received repeated echocardiogram and electrocardiogram examinations during follow-ups. Seven patients had mildly abnormal electrocardiogram results, including sinus tachycardia (in 3 cases), PR prolongation (in 1 case), QT interval prolongation (in 3 cases) and changes in ST segment and T waves (in 2 cases) (some patients had multiple abnormal electrocardiogram changes). Two patients had mild interventricular septal hypertrophy. In total, 9 patients (29%, 9/31) showed mild abnormal electrocardiograms and/or echocardiograms. MmD (4/9, 44.4%) was found to be the most frequent pathological diagnosis in patients with mild cardiac abnormalities. Gene variations in *RYR1* (2 cases), *TTN* (2 cases) and *TNNT1* (1 Case) were identified in 5 of these patients.

During the follow-up, three patients (3/31) had slurred speech and mild difficulty swallowing large chunks. Three patients were underweight (below the 3rd centile), and 2 were overweight (above the 97th centile). Twenty-three patients (23/31) were studying at normal public school, and no one complained of learning difficulty. Eight patients were not in school due to the following reasons: five of the patients were under school age, and the remaining three did not go to school due to walking difficulties. No malignant hyperthermia was noted in the patients with *RYR1* variations.

## Discussion

Congenital myopathies are a rare group of muscle diseases with clinical and genetic heterogeneity [3]. In this study, we analyzed the clinical, genetic and pathological data of patients with infancy-onset congenital myopathies. The most common presentations in our patients were generalized muscle weakness and hypotonia as well as proximally predominant limb weakness combined with axial muscle involvement, which was consistent with other studies [10, 11]. However, the frequency of neonatal bulbar involvement and respiratory failure (13.8%) was significantly lower than that in other studies, which may contribute to the low infant mortality in this cohort. Colombo et al. reported that 30.4%(38/125) of patients required respiratory support at birth, and that 25.2% required nasogastric feeding at birth [10]. Another study has reported a mortality rate for congenital myopathies of 8% (5/66) with 4 out of 5 deaths occurring within the first 2 months of life [11]. Compared to these studies, none of our patients died during infancy. There are several reasons for this discrepancy. The discrepancy is mainly due to the underlying genes as there is a different prevalence of myopathy-causing genes among patients from different ethnic groups [19, 20]. It has been reported that severe neonatal bulbar and respiratory muscle involvement occurs particularly in severe NM (most likely *ACTA1*-, *NEB*- or *KLHL40*-related) and *MTM1*-related myotubular myopathy [1, 12, 25, 26]. *XMTM1* gene variations are recognized as the most lethal cause of congenital myopathy in infancy [12]. In Colombo’s cohort, the variations in *ACTA1, MTM1 or KLHL40* accounted for a large portion of the patients with severe bulbar involvement and caused a 12% death rate, mainly within the first year. Comparatively, only four *ACTA1* pathogenic variations were identified in our patients, and *MTM1* or *KLHL40* pathogenic variations were not found. Moreover, sampling bias should be noted. The present study was performed at a single paediatric centre in China. Most of our patients came to us from all across mainland China, and the patients with lethal complications during the perinatal period might be absent from the present study. There may have been a few patients who died of severe congenital myopathies before visiting us.

In contrast to many other neuromuscular diseases, cardiac involvement represents a major cause of morbidity and mortality [13]. Cardiac involvement is not an important issue in most cases of congenital myopathies [3]. Congenital myopathies are generally associated with a mild cardiac phenotype [18], and lethal cases have rarely been reported [27, 28]. None of our patients complained of cardiac symptoms, and only mildly abnormal changes on electrocardiograms and/or echocardiograms were detected. The mildness of cardiac involvement in our patients was consistent with that in previous reports [18, 27]. *TTN* and *MYH7* are the most frequent genes related to severe cardiac involvement in the literature [29], whereas *ACTA1*, *RYR1*, *TPM2*, *FLNC* and *SPEG* have occasionally been reported [15, 17, 27, 30]. Although 2 out of 3 *TTN* patients were found to have mild cardiac abnormalities in our cohort, it was difficult to analyze the severity of cardiac involvement between different gene variations due to the small sample size. Considering the relatively high frequency (29%, 9/31) of mild cardiac involvement in our cohort, our observation suggested that cardiac involvement in congenital myopathies may be underestimated. The issue of cardiac involvement in congenital myopathies and its relationship with different gene variations needs further study.

Core myopathy and NM were the most frequent pathologies in our cohort, and *RYR1* was the most common associated gene, similar to previous reports but with varied rates between studies [10, 11]. Heterozygous dominant *RYR1* variations were pathologically associated with CCD, and recessive *RYR1* variations were associated with different pathological changes, including MmD, CNM and CFTD, which was consistent with previous reports [7, 9, 31, 32]. Patients with *RYR1* variations have a wide clinical spectrum with variable severity [33, 34]. It has been reported that dominant *RYR1* variations are associated with milder phenotypes, and patients with recessive *RYR1* variations have earlier onset, more weakness and functional limitations [32]. It was difficult to fully discriminate the presentations of dominant and recessive *RYR1* variations due to the small sample size in our study. However, different from previous reports, the motor ability of the patients with dominant *RYR1* variations was worse than that of patients with recessive variations in our follow-up patients. We believe that the clinical heterogeneity of *RYR1* requires more research.

Novel complex heterogeneous *TNNT1* variations were identified in one of our patients (Pt 9). *TNNT1* was first identified in Order Amish patients with NM as the causing gene [20]. The *TNNT1* c.505G > T variation has a carrier frequency of 6.5% within Old Order Amish settlements of North America [35]. In their first months of life, afflicted Amish infants have tremors with hypotonia and mild contractures of the shoulders and hips followed by progressive muscle weakness, atrophy and contractures. Early respiratory failure and striking stiffness of the cervical spine usually cause death in the second year [20, 35]. The prominent axial muscle involvement and multiple contractures in the hips, shoulders, elbows and knees in Pt 9 were similar to those of Amish patients. However, Pt 9 presented with hypotonia and motor delay from the age of 6 months with a normal neonatal period and without tremors throughout the clinical course. Pt 9 was still alive at 11 years old with supported walking, severe scoliosis and restrictive respiratory insufficiency. The symptoms of Pt 9 were much milder than that of Amish patients. In addition, most of the reported *TNNT1* variations were homozygous null variations [36], whereas one paternal frameshift variation (c.353delC; p. Thr118MetfsTer16) and one maternal missense variation (c.1A > G; p. Met1Val) were identified in our patients, which expanded the genotype of *TNNT1*.

## Conclusion

The present study expanded the clinical and genetic spectrum of congenital myopathies. First, we reported 18 novel variations in 6 myopathy-causing genes, including *RYR1, NEB, ACTA1, DNM2, TTN* and *TNNT1, and* we described the clinical discrepancy between our patients and previous reports, especially in *RYR1-* and *TNNT1-* related myopathy. Second, in addition to routine monitoring of muscular complications, ambulatory loss and respiratory failure, our follow-up data provided more details of nonmuscle involvements in patients with congenital myopathies. The necessity of cardiac function evaluation is emphasized, even in patients with static courses.

## Supplementary Information

**Additional file 1.**

**Additional file 2.**

## Data Availability

The datasets used and/or analyzed during the present study are available from the corresponding author on reasonable request. Novel gene variations have been submitted to the ClinVar database (https:// www.ncbi.nlm.nih.gov/clinvar/).

## Abbreviations

AD: Autosomal dominant
AR: Autosomal recessive
ATPase: Adenosine triphosphatase
CCD: Central core disease
CFTD: Congenital fibre type disproportion
CNM: Centronuclear myopathy
COX: Cytochrome c oxidase
FVC: Forced vital capacity
H&E: Haematoxylin and eosin
MGT: Modified Gomori trichrome
MmD: Multiminicore disease
NADH-TR: Nicotinamide adenine dinucleotide dehydrogenase-tetrazolium reductase
NM: Nemaline myopathy
PAS: Periodic acid-Shiff
SDH: Succinate dehydrogenase
WES: Whole exome sequencing
