# Supplementary material for: Clinical and genetic features of infancy-onset congenital myopathies from a Chinese paediatric centre
Source: BMC Pediatr. 2022 Jan 26;22:65. doi: 10.1186/s12887-021-03024-0 (PMC8790871; doi:10.1186/s12887-021-03024-0)
Supplement: Supplementary file 1 — Additional file 1. [file 12887_2021_3024_MOESM1_ESM.doc]

Gene list comprising the panel for hereditary muscle disease (the 169 genes)

| ABHD5 | ACADVL | ACTA1 | ACVR1 | AGK | AGL | AGRN | ALG13 |
| --- | --- | --- | --- | --- | --- | --- | --- |
| ALG14 | ALG2 | ANO5 | ATP2A1 | ATP5A1 | B3GALNT2 | B3GNT1 | BAG3 |
| BIN1 | CABC1 | CACNA1A | CACNA1S | CAPN3 | CAV3 | CCDC78 | CFL2 |
| CHAT | CHKB | CHRNA1 | CHRNB1 | CHRND | CHRNE | CHRNG | CHST14 |
| CLCN1 | CNBP | SLC28A2 | CNTN1 | COL12A1 | COL6A1 | COL6A2 | COL6A3 |
| COLQ | CPT2 | CRYAB | DAG1 | DARS | DES | DMD | DMPK |
| DNAJB6 | DNM2 | DOK7 | DOLK | DPAGT1 | DPM1 | DPM2 | DPM3 |
| DUX4 | DYSF | ECEL1 | EMD | ENO3 | ETFA | ETFB | ETFDH |
| FHL1 | FKBP14 | FKRP | FKTN | FLNC | GAA | GBE1 | GFPT1 |
| GMPPB | GNE | GTDC2 | GYG1 | GYS1 | HADHB | HSPG2 | ISCU |
| ISPD | ITGA7 | ITGA9 | KBTBD13 | KCNA1 | KCNE3 | KLHL40 | KLHL41 |
| KLHL9 | LAMA2 | LAMB2 | LAMP2 | LARGE | LDB3 | LDHA | LMNA |
| LPIN1 | LRP4 | MAMLD1 | MATR3 | MEGF10 | MSTN | MTM1 | MTMR14 |
| MUSK | MYBPC1 | MYBPC3 | MYF6 | MYH14 | MYH2 | MYH7 | MYOT |
| NDUFB3 | NEB | PABPN1 | PFKM | PGAM2 | PGK1 | PGM1 | PHKA1 |
| PIEZO2 | PLEC | PLOD1 | PLOD2 | PLOD3 | PNPLA2 | POLG2 | POMGNT1 |
| POMK | POMT1 | POMT2 | PRKAG2 | PTPLA | PTRF | PUS1 | PYGM |
| RAPSN | RBCK1 | RYR1 | SCN4A | SEPN1 | SGCA | SGCB | SGCD |
| SGCE | SGCG | SGK196 | SIL1 | SLC16A2 | SLC22A5 | SLC25A20 | SMCHD1 |
| SYNE1 | SYNE2 | TCAP | TIA1 | TK2 | TMEM43 | TMEM5 | TNNT1 |
| TNPO3 | TOR1AIP1 | TPM2 | TPM3 | TRAPPC11 | TRIM32 | TTN | VCP |
| YARS2 |  |  |  |  |  |  |  |
