# Supplementary material for: Clinical and genetic features of infancy-onset congenital myopathies from a Chinese paediatric centre
Source: BMC Pediatr. 2022 Jan 26;22:65. doi: 10.1186/s12887-021-03024-0 (PMC8790871; doi:10.1186/s12887-021-03024-0)
Supplement: Supplementary file 2 — Additional file 2. [file 12887_2021_3024_MOESM2_ESM.doc]

**Supplement 2**. genetic data of patients with congenital myopathies

| Case | Gene | Variations | Protein | PMID | ClinVar Allele ID | Classification of variation |
| --- | --- | --- | --- | --- | --- | --- |
| 1 | *RYR1* | c.14596A>G^‡^ | p.(Lys4866Gln) | 25331388 |  | missense |
| 2 | *RYR1* | c.7111G>A^‡^ | p.(Glu2371Lys) | 29293505 |  | missense |
| 3^#^ | *RYR1* | c.14678 G>A^‡^ | p.(Arg4893Gln) | 12565913 | 361051 | missense |
| 4^#^ | *RYR1* | c.14741G>C^‡^ | p.(Arg4914Thr) | 12565931 |  | missense |
| 5 | *TTN* | c.85818T>A | p.(Tyr28606Ter) |  | 858777 | missense |
|  |  | c.102798_102800del | p.(Asn34266del) |  |  | deletion |
| 6 | *RYR1* | c.3880G>T | p.(Val1294Phe) |  |  | missense |
|  |  | c.14473C>T^‡^ | p.(Arg4825Cys) | 20301565 | 76893 | missense |
| 7^#^ | *RYR1* | c.658C>T | p.(Arg220Cys) |  |  | missense |
|  |  | c.4715T>C | p.(Met1572Thr) |  |  | missense |
| 8 | *RYR1* | c.4454G>A | p.(Ser1485Asn) |  |  | missense |
|  |  | c.3494G>A^‡^ | p.(Gly1165Asp) | 21911697 |  | missense |
| 9 | *TNNT1* | c.1A>G | p.? |  |  | missense |
|  |  | c.353delC | p. (Thr118Metfs  Ter16) |  |  | frameshit |
| 10 | *NEB* | c.18808C>T^‡^  c.2311-2A>C^‡^ | p.(Arg6270Ter)  p. ? | 25205138  32222963 | 541657 | Missense  splice |
| 12 | *TTN* | c.2099_2106dup | p.(Ala703LysfsTer3) |  | 858778 | frameshift |
|  |  | c.107377+1G>A^‡^ | p.? | 25589632 | 193884 | splice |
| Case | Gene | Variations | Protein | PMID | ClinVar Allele ID | Classification of variation |
| 13 | RYR1 | c.6823G>A | p. (Val2275Met) |  |  | missense |
|  |  | c.2044C>G | p.(Arg682Gly) |  |  | missense |
| 14 | *TTN* | c.95341C>T^‡^ | p.(Arg31781Ter) | 25163546 |  | missense |
|  |  | c.32312-1G>A | p.? |  | 858790 | splice |
| 15 | *DNM2* | c.1893+1G>A | p.? |  | 858791 | splice |
| 16 | *DNM2* | c.1856C>T^‡^ | p.(Ser619Leu) | 32860008 | 22324 | missense |
| 17 | *RYR1* | c.12536G>A^‡^ | p.(Arg4179His) | 21062345 |  | missense |
|  |  | c.1675dup | p.(Ile559AsnfsTer11) |  | 858780 | frameshift |
| 18 | *RYR1* | c.3523G>A^‡^ | p.(Glu1175Lys) | 25635128 |  | missense |
|  |  | c.7330C>T | p.(Gln2444Ter) |  | 858786 | missense |
| 34 | *RYR1* | c.14447A>G^‡^ | p.(Asp4816Gly) | 23553484 | 858787 | missense |
| 35 | *RYR1* | c.14582G>A^‡^ | p.(Arg4861His) | 25521991 |  | missense |
| 36^#^ | *NEB* | c.3567+1G>A | p.? |  | 135187 | splice |
|  |  | c.6734dupA | p. (Thr2246Aspfs  Ter8) |  |  | frameshift |
| 37 | *NEB* | c.19944G>A^‡^ | p.？ | 25205138 | 237087 | missense |
|  |  | c.6029del^‡^ | p. (Ile2010Thrfs  Ter14) | 32222963 |  | frameshift |
|  |  |  |  |  |  |  |
|  |  |  |  |  |  |  |
| Case | Gene | Variations | Protein | PMID | ClinVar Allele ID | Classification of variation |
| 38 | *NEB* | c.7818delG^‡^ | p. (Met2606Ilefs  Ter13) | 32222963 |  | frameshift |
|  |  | c.24579G>A ^‡^ | p.？ | 24725366 | 511326 | missense |
| 39 | *ACTA1* | c.400A>G^‡^ | p.(Met134Val) | 10508519 | 1019319 | missense |
| 40 | *ACTA1* | c.515C>G^‡^ | p.(Ala172Gly) | 12921789 |  | missense |
| 41 | *ACTA1* | c.402G>T | p.(Met134Ile) |  | 858775 | missense |
| 42 | *ACTA1* | c.109G>T^‡^ | p.(Val37Leu) | 25326635 |  | missense |

Note: Reference transcript of different genes: *RYR1*, NM_000540.2; *NEB*, NM_001164507.1; *DNM2*, NM_001005360.2; *ACTA1*, NM_001100.3; *TNNT1*, NM_001126132.1; *TTN*, NM_001267550.1. PMID: PubMed Unique Identifier.

Abbreviations: #, positive family history; ‡, previously reported; /, data unavailable;
